# Antimicrobial Activity of Five Apitoxins from *Apis mellifera* on Two Common Foodborne Pathogens

**DOI:** 10.3390/antibiotics9070367

**Published:** 2020-06-30

**Authors:** Alexandre Lamas, Vicente Arteaga, Patricia Regal, Beatriz Vázquez, José Manuel Miranda, Alberto Cepeda, Carlos Manuel Franco

**Affiliations:** 1 Laboratorio de Higiene Inspección y Control de Alimentos, Departamento de Química Analítica, Nutrición y Bromatología, Universidad de Santiago de Compostela, 27002 Lugo, Spain; alexandre.lamas@usc.es (A.L.); patricia.regal@usc.es (P.R.); beatriz.vazquez@usc.es (B.V.); josemanuel.miranda@usc.es (J.M.M.); alberto.cepeda@usc.es (A.C.); 2 Laboratorio de Microbiología, Escuela de Ciencias Agrícolas y Ambientales (ECAA) Pontificia Universidad Católica del Ecuador, Sede Ibarra, Ibarra 100112, Ecuador; varteaga@pucesi.edu.ec

**Keywords:** apitoxin, antimicrobial resistance, natural antimicrobial compounds, foodborne pathogens, *Salmonella*, *Listeria monocytogenes*

## Abstract

Antimicrobial resistance is one of today’s major public health challenges. Infections caused by multidrug-resistant bacteria have been responsible for an increasing number of deaths in recent decades. These resistant bacteria are also a concern in the food chain, as bacteria can resist common biocides used in the food industry and reach consumers. As a consequence, the search for alternatives to common antimicrobials by the scientific community has intensified. Substances obtained from nature have shown great potential as new sources of antimicrobial activity. The aim of this study was to evaluate the antimicrobial activity of five bee venoms, also called apitoxins, against two common foodborne pathogens. A total of 50 strains of the Gram-negative pathogen *Salmonella enterica* and 8 strains of the Gram-positive pathogen *Listeria monocytogenes* were tested. The results show that the minimum inhibitory concentration (MIC) values were highly influenced by the bacterial genus. The MIC values ranged from 256 to 1024 µg/mL in *S. enterica* and from 16 to 32 µg/mL in *L. monocytogenes.* The results of this study demonstrate that apitoxin is a potential alternative agent against common foodborne pathogens, and it can be included in the development of new models to inhibit the growth of pathogenic bacteria in the food chain.

## 1. Introduction

The discovery and development of antimicrobial agents in the first half of the 20th century created a new paradigm. Since that time, common infections that would have caused death have become treatable with antibiotics, saving millions of lives. At first, the use of antimicrobials was generalized, and they were used to treat both human and animal infections [1]. Antimicrobials were, and still are, used for zootechnical purposes in farm animals [1]. Soon after the discovery of antibiotics, the phenomenon of antimicrobial resistance was addressed. In his 1945 Nobel Prize lecture, Sir Alexander Fleming stated that “there is the danger that the ignorant man may easily under dose himself and by exposing his microbes to non-lethal quantities of the drug make them resistant” [2]. This warning has become a reality; antimicrobial resistance is a global public health problem [3]. It is estimated that antimicrobial resistance in common bacterial infections is responsible for 700,000 deaths worldwide each year, with the potential to reach millions of deaths per year by 2050. In the European Union alone, there are 25,000 deaths each year related to antimicrobial resistance. In addition, antimicrobial resistance causes serious economic damage, estimated at $1.5 trillion in health care costs and lost productivity [4]. Multidrug-resistant bacteria are also a serious problem in the food production chain [5]. Studies in recent years have found a large number of strains of multidrug-resistant foodborne pathogens. The use of antibiotics in production animals and the resultant selective pressure on the environmental microbiota together constitute one of the main causes of the current exponential increase in antimicrobial resistance.

Therefore, a current research priority is the search for and discovery of alternatives to conventional antibiotics. The three principal research strategies can be classified as (i) naturally occurring alternatives, (ii) synthetic designs, and (iii) biotechnology-based strategies [6]. The most common naturally occurring alternatives are bacteriocins, bacteriophages, and antimicrobial peptides (AMPs) [6]. Of these, AMPs have received great attention from the research community in recent years. These naturally derived molecules are part of the innate immune system in both prokaryotic and eukaryotic cells; their main advantages with respect to other natural alternatives are their broad-spectrum activity and lack of susceptibility to resistance development [6,7,8]. AMPs’ mode of action is based on the permeabilization of bacterial membranes and the formation of cytotoxic pores, but they can also inhibit nucleotides, proteins, and cell wall biosynthesis [9]. Practical studies have demonstrated that AMPs are a promising alternative for combating common foodborne pathogens such as *Salmonella*, *L. monocytogenes,* and *Staphylococcus aureus* [8,10,11]. The water-soluble peptide melittin from honeybee venom is one of these promising AMPs. Melittin has demonstrated both antimicrobial and antiviral activity in in vitro studies [12,13]. Melittin is a 26 amino acid cationic linear peptide with an N-terminal hydrophobic region, a C-terminal hydrophilic region, and asymmetrical distribution of polar and nonpolar amino acid residues. This suggests an amphipathic nature in α-helical conformation that makes melittin a membrane-active molecule. Due to its nature, melittin exerts antimicrobial activity by destabilizing the bacterial membrane and causing pore formation, which induces a loss of osmotic balance and, ultimately, cell lysis. Specifically, the perpendicular orientation of melittin to the cell membrane causes its insertion, peptide aggregation, and the bending of lipids, resulting in the leakage of cytoplasmic contents [14,15,16]. However, honeybee venom, or apitoxin, is also composed of other peptides such as adolapin, apamin, and MCD peptide, and enzymes such as phospholipase A2 and hyaluronidase [16]. Although melittin is the most bioactive component of apitoxin, its bioactivity is enhanced by other components of bee venom [17]. In this sense, it has been demonstrated that melittin and phospholipase A2 have synergistic activity. Melittin exposes membrane phospholipids through pore formation to the catalytic site of phospholipase A2 [14]. Although the antimicrobial properties of melittin have been studied in depth, only limited studies have evaluated the antimicrobial ability of apitoxin, and very few strains were included [12,18]. It is therefore necessary to determine whether the apitoxins obtained in different geographic locations and tested in different studies show similar inhibition values. It is also important that these types of studies include a large collection of wild strains to increase the significance of the data obtained.

Therefore, the aim of this study is to evaluate the antimicrobial activity and determine the minimum inhibitory concentration (MIC) of five apitoxins obtained from apiaries located in different parts of Ecuador on a large collection of wild-strain foodborne pathogens. For this purpose, 50 *Salmonella* strains belonging to different serotypes and subspecies and 8 *L. monocytogenes* strains were included in this study. These pathogens were selected because *Salmonella* spp. and *L. monocytogenes* are two of the main foodborne pathogens in the European Union, with 91,662 and 2480 confirmed cases of human infections in 2017, respectively. Moreover, by including these two pathogens, we tested both Gram-positive and Gram-negative bacteria.

## 2. Results

The amount of apitoxin collected each time was between 29 and 40 mg. The amount collected from apitoxin 1 was 34.33 ± 2.98 mg, from apitoxin 2 was 36.55 ± 1.46 mg, from apitoxin 3 was 37.25 ± 4.95 mg, from apitoxin 4 was 39.66 ± 0.67 mg, and from apitoxin 5 was 39.66 ± 0.78 mg. There were significant differences (*p* < 0.05) in the amounts between apitoxin 1 and apitoxins 4 and 5. There were no significant differences (*p* < 0.05) in the concentration of melittin in the apitoxins tested in this study, with values around 129 µg/mL. The five tested apitoxins showed antimicrobial activity against all *S. enterica* and *L. monocytogenes* strains included in this study. In *S. enterica*, the MIC values ranged between 256 and 1024 µg/mL (Table 1), but most of the strains showed an MIC value of 512 µg/mL. The lowest inhibitory concentration in Apitoxins 1 and 4 was 256 µg/mL, and four and three strains, respectively, showed an MIC value of 1024 µg/mL. On the other hand, apitoxin 5 showed the most strains in which growth was inhibited at 256 µg/mL.

The value of MIC_90_ and MIC_50_ for *S. enterica* was 512 µg/mL for four of the apitoxins tested. However, the MIC_90_ of apitoxin 1 was 1024 µg/mL, which could indicate lower antimicrobial activity. There were no significant differences (*p* > 0.05) in MIC values between the five apitoxins tested. It is also remarkable that four *S.* Infantis strains showed an MIC of 256 µg/mL in four of the apitoxins tested. This indicates a higher susceptibility of those strains to apitoxin in comparison with the other strains of *S.* Infantis. In this sense, there were significant differences (*p* < 0.05) in resistance results between *S.* Infantis and the other strains of *S. enterica* subspecies *enterica*. On the other hand, the strain *S. enterica* subspecies *salamae* SA3 showed higher values, with an MIC of 1024 µg/mL in the five apitoxins tested. In fact, *S. enterica* subspecies *salamae* was significantly more resistant (*p* < 0.05) than *S. enterica* subspecies *arizonae* or *S. enterica* subspecies *enterica*.

In the case of *L. monocytogenes,* the MIC values observed were lower than those found in *S. enterica* strains, ranging between 16 and 32 µg/mL (Table 2). There were differences in the MIC_50_ of the apitoxins used in this study. For apitoxins 1, 2, and 4, the MIC_50_ was 16 µg/mL, and for apitoxins 3 and 5, it was 32 µg/mL. All apitoxins had an MIC_90_ of 32 µg/mL. It is also remarkable that all tested *L. monocytogenes* strains showed an MIC of 32 µg/mL with apitoxin 5. The MIC value of *L. monocytogenes* was significantly lower (*p* < 0.05) than that of *Salmonella* spp.

## 3. Discussion

The discovery and evaluation of new and natural antimicrobial substances is one of the main strategies to decrease the use of antibiotics and avoid the increase in multidrug-resistant strains [6]. In the last several decades, compounds isolated from natural products have shown promising activity against resistant bacteria [19]. In this sense, venoms have been shown to be composed of various substances, such as antimicrobial peptides, with high inhibitory activity [15,20,21]. In this study the antimicrobial activity of bee venom was tested. Different works have observed that the main components of apitoxin, such as pure melittin and phospholipase A, have high antimicrobial activity against different bacterial pathogens [15]. However, a very limited number of studies have tested the inhibitory capacity of pure apitoxin in bacteria, and the information available on foodborne pathogens is currently insufficient [22]. The results of this study show significant differences in MIC values between *Salmonella* (256–1024 µg/mL) and *L. monocytogenes* (16–32 µg/mL) strains. A previous study that evaluated the activity of commercially available apitoxin against oral bacteria such as *Enterococcus faecalis* and *Streptococcus salivarius* found MIC values between 20 and 40 µg/mL [18], very similar to the results observed in *L. monocytogenes* in this study. Additionally, a recent study found an MIC of 7.2 µg/mL in strains of Gram-positive *Staphylococcus aureus* bacteria [12]. In the same way, Picoli et al. [23] observed that *S. aureus* had lower MIC values (6–7 µg/mL) than Gram-negative *Escherichia coli* (40–42.5 µg/mL) and *Pseudomonas aeruginosa* (65–70 µg/mL) bacteria for the AMP of bee venom melittin. These differences can be related to structural differences between Gram-positive and Gram-negative bacteria. In this sense, it has been suggested that melittin can penetrate the peptidoglycan layer of Gram-positive bacteria more easily than the membrane of Gram-negative bacteria, which is protected by a layer of lipopolysaccharides [13,14]. In the same way, the phospholipase A2 present in apitoxin causes phospholipid membrane degradation, resulting in cell death [13,14]. However, the outer membrane in Gram-negative bacteria reduces the efficacy of phospholipase A2 by reducing the interaction of this enzyme with the cytoplasmic membrane [16]. Therefore, the combination of apitoxin with other substances that disrupt the outer membrane of Gram-negative bacteria could increase the antimicrobial activity of apitoxin. One of the main advantages of the present study in comparison with the studies previously described is the number of strains included. *Salmonella* spp. are composed of more than 2600 serotypes and six subspecies, which differ in their pathogenicity [24]. The results of this study show that the observed MIC values were very stable through the *Salmonella enterica* species, but there were some significant differences between some subspecies and serotypes of *Salmonella enterica* subspecies *enterica*. In addition, the differences observed between the five apitoxins were not due to different concentrations of melittin, as no significant differences were observed between them.

## 4. Materials and Methods

### 4.1. Apitoxin Collection

**Figure 1:**
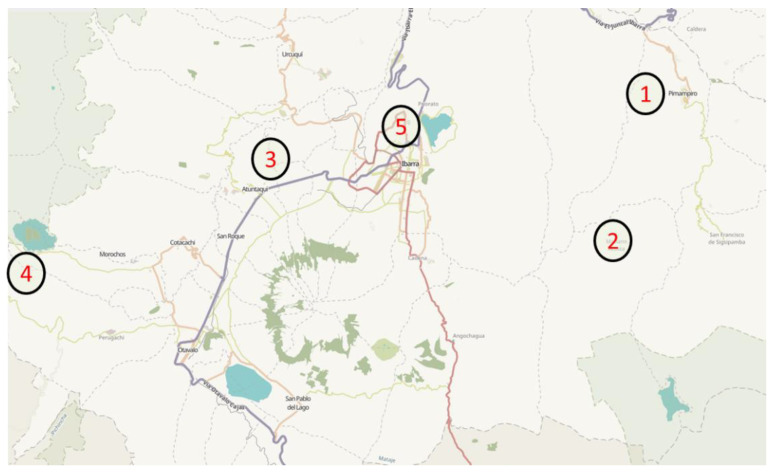
Geographic location of five apiaries in the province of Imbabura (Ecuador). 1: Apiary El Inca; 2: Apiary Caranqui; 3: Apiary Clatura; 4: Apiary Cotacachi; and 5: Apiary ECAA.

Bee venom, or apitoxin, was collected from 5 *Apis mellifera* apiaries in Ecuador: El Inca (apitoxin 1), Apiary Caranqui (apitoxin 2), Apiary Clatura (apitoxin 3), Apiary Cotacachi (apitoxin 4), and Apiary ECAA (apitoxin 5) (Figure 1). The collections were made between 11:00 and 13:00 from 9 January to 28 May 2016, with an interval of 21 days between collections, until 5 collections per apiary and hive were completed. Apitoxin was collected by using an electric stimulus, as previously described [25]. Briefly, when bees land on a woven copper wire located inside the beehive, an electric stimulus is applied, causing the release of bee venom without killing the bees. The bee venom is collected on glass slides, where the apitoxin crystallizes. The glass slides are transported to the laboratory, where the crystallized bee venom is detached with a scraper, collected in microtubes, and weighed. This crude apitoxin was used in subsequent analyses.

### 4.2. Mellitin Determination of Apitoxin by HPLC-UV

The melittin content of the 5 apitoxin samples was determined according the method developed by Rybak-Chmielewska and Szczêsna [26] with some modifications. A melittin standard with 96.5% purity was obtained from Sigma-Aldrich (Germany). Briefly, 5 mg of apitoxin was mixed with 5 mL of ultrapure water and sonicated for 5 min, and the liquid was filtered through a 0.45 μm polytetrafluoroethylene syringe filter and collected in an amber glass vial. A volume of 5 µL of 85% phosphoric acid was added to the vial. The samples were analyzed by HPLC in a Jasco LC-Net II/ADC (Jasco, Spain) coupled with a UV-2070 detector (Jasco). A Machery-Nagel C-18 column with a length of 250 mm, internal diameter of 4 mm, and particle size of 5 µm was used. Gradient chromatography was performed by the linear method with 5–80% of eluent (acetonitrile in 20% phosphoric acid) for 45 min with flow velocity of the moving phase at 1 mL·min^−1^. Melittin was identified at 220 nm wavelength. The data were collected through the use of Chrom NAV software (Jasco).

### 4.3. Salmonella and L. monocytogenes Strains

A total of 50 *S. enterica* and 8 *L. monocytogenes* strains, including culture collection strains *Salmonella* CECT 4395 and *L. monocytogenes* CECT 934, were used in this study. The rest of the *Salmonella* strains were isolated in our laboratory from poultry farms within the framework of the national *Salmonella* control plan and from chicken meat according ISO 6579:2017 [27]. All *Salmonella* strains were serotyped using the Kauffman–White typing scheme for the detection of somatic (O) and flagellar (H) antigens with standard antisera (Bio-Rad Laboratories, Irvine, CA, USA). The rest of the *L. monocytogenes* strains were isolated in our laboratory from food products (rabbit meat, cheese, fish products) by routine analysis for the food industry according to ISO 11290-1:2017 [28]. *Salmonella* strains were kept at −20 °C in Tryptic Soy Broth (TSB; Oxoid, Basingstoke, UK) supplemented with 20% glycerol, and *L. monocytogenes* strains were kept in Brain Heart Infusion (PanReac AppliChem, Barcelona, Spain) supplemented with 20% glycerol until use.

### 4.4. Determination of Minium Inhibitory and Biocidal Concentrations

The MIC of the 5 apitoxins included in this study was determined according Clinical and Laboratory Standards Institute (CLSI) guidelines by using the broth microdilution method. Briefly, an initial stock of 4096 µg/mL of each apitoxin was prepared. Dilutions of apitoxin in Mueller–Hinton agar from 2048 µg/mL to 2 µg/mL were made. *Salmonella* and *L. monocytogenes* strains were grown in nutrient agar (PanReac, AppliChem, Spain) for 24 h at 37 °C. Isolated colonies were used to obtain a saline suspension of 0.5 McFarland equivalent to 10^8^ colony-forming units (CFU)/mL. This suspension was diluted to 1:20 to obtain a final concentration of 10^6^ CFU/mL. The broth volume in a 96-well microtiter plate was 0.1 mL, and 0.01 mL of the diluted bacterial suspension was inoculated to a final bacterial concentration of 10^4^ CFU/mL. The 96-well microtiter plates were incubated for 24 h at 37 °C, and the MIC value of each strain with each apitoxin was determined. The MIC was defined as the lowest concentration of antimicrobial agent that completely inhibited the visual growth of the organism in the wells.

### 4.5. Stastitical Analysis

GraphPad Prism 8 (GraphPad, San Diego, CA, USA) was used in this research for statistical analysis. Chi-squared tests were performed to evaluate the differences between the 5 apitoxins tested and between genera, subspecies, and serotypes. Analysis of variance (one-way ANOVA) and Tukey’s honestly significant difference test (*p* < 0.05) were used to determine the differences between the amounts of apitoxin collected from the apiaries.

## 5. Conclusions

This study increases the information available on the antimicrobial capacity of apitoxin against foodborne pathogens. The results demonstrate that apitoxin is a potential alternative agent to inhibit the growth of common foodborne pathogens in the food chain at low concentrations, especially in *L. monocytogenes* strains. Therefore, apitoxin can potentially be used alone as an alternative to common antimicrobials or even in combination with them to enhance the antimicrobial activity of both substances. Future studies should be focused on developing new models to apply this substance at different steps of the food chain in order to translate in vitro results to real-life applications.

## Figures and Tables

**Table 1:** Minimum inhibitory concentrations (MICs) of five apitoxins tested in 50 *Salmonella* strains isolated from poultry.

			MIC (µg/mL)				
Strain	Source	Code	Apitoxin 1	Apitoxin 2	Apitoxin 3	Apitoxin 4	Apitoxin 5
*S.* Anatum	PF	A1	512	256	256	512	512
*S.* Anatum	PF	A6	512	512	512	512	512
*S.* Anatum	PF	A15	512	512	512	512	512
*S. enterica* subspecies *arizonae*	PF	AZ1	512	256	256	512	512
*S. enterica* subspecies *arizonae*	PF	AZ6	512	256	256	512	256
*S. enterica* subspecies *arizonae*	PF	AZ12	256	256	256	512	512
*S. enterica* subspecies *arizonae*	PF	AZ16	1024	512	512	512	512
*S. enterica* subspecies *arizonae*	PF	AZ20	512	256	512	512	512
*S. enterica* subspecies *arizonae*	PF	AZ21	512	512	256	256	256
*S.* Bardo	PF	B2	512	512	512	512	512
*S.* Bardo	PF	B3	512	512	512	512	512
*S.* Bredeney	PF	BR1	1024	512	512	512	512
*S.* Dabou	PF	DA1	512	512	512	512	256
*S.* Drac	PF	DC4	1024	512	512	512	512
*S.* Enteritidis	CK	ET1	512	512	256	256	256
*S.* Enteritidis	PF	ET2	512	512	256	256	512
*S.* Infantis	PF	I1	256	256	256	512	256
*S.* Infantis	PF	I2	256	256	256	512	256
*S.* Infantis	PF	I3	256	256	256	512	256
*S.* Infantis	PF	I4	256	256	256	1024	256
S. Infantis	PF	I7	512	512	512	256	256
*S.* Infantis	PF	I12	512	512	512	512	512
S. Infantis	PF	I11	512	512	512	512	512
*S.* Infantis	PF	I18	512	512	512	256	512
*S.* Isangi	PF	IG1	512	512	512	512	512
*S.* Isangi	PF	IG9	512	512	512	512	512
*S.* Montevideo	PF	M1	512	512	512	512	512
*S.* Mbandaka	PF	MB1	512	512	512	512	256
*S.* Ndolo	PF	ND1	512	512	512	512	512
*S.* Ndolo	PF	ND2	512	512	512	512	512
*S.* Ndolo	PF	ND5	512	512	512	256	256
*S.* Newport	PF	N1	512	512	512	512	512
*S.* Newport	PF	N6	512	512	512	512	512
*S.* Rissen	PF	R1	512	512	512	512	256
*S. enterica* subspecies *salamae*	PF	SA1	512	512	512	1024	512
*S. enterica* subspecies *salamae*	PF	SA2	1024	512	512	1024	512
*S. enterica* subspecies *salamae*	PF	SA3	1024	1024	1024	1024	1024
*S.* Seftenberg	PF	S1	512	512	512	512	512
*S.* Stanleyville	PF	ST1	512	512	512	512	512
*S.* Thompson	PF	TM1	1024	512	512	512	512
*S.* Typhimurium	CK	T2	512	256	512	512	256
*S.* Typhimurium	CK	T3	512	512	256	512	512
*S.* Typhimurium	PF	T6	512	512	512	512	512
*S.* Typhimurium	PF	T10	512	512	256	512	256
*S.* Typhimurium	PF	T12	512	512	512	512	256
*S.* Typhimurium	PF	T13	512	256	256	512	256
*S.* Typhimurium	PF	T18	512	512	512	512	512
*S.* Typhimurium	PF	T21	256	512	256	256	256
*S.* Typhimurium	PF	T24	512	512	512	512	512
*S.* Typhimurium	CC	CECT 4395	512	512	512	512	512
			n (%)	n (%)	n (%)	n (%)	n (%)
MIC (µg/mL)		256	6 (12%)	11 (22%)	15 (30%)	7 (14%)	17 (34%)
		512	38 (76%)	38 (76%)	34 (68%)	39 (78%)	33 (64%)
		1024	6 (12%)	1 (2%)	1 (2%)	4 (8%)	1 (2%)

CC, culture collection; CK, chicken meat; PF, poultry farm.

**Table 2:** Minimum inhibitory concentrations of five apitoxins tested in eight *L. monocytogenes* strains isolated from foodstuff.

			MIC (µg/mL)				
Strain	Source	Code	Apitoxin 1	Apitoxin 2	Apitoxin 3	Apitoxin 4	Apitoxin 5
*L. monocytogenes*	RM	LHICA 1	16	16	32	16	32
*L. monocytogenes*	RM	LHICA 2	16	16	32	16	32
*L. monocytogenes*	CH	LHICA 3	32	16	32	32	32
*L. monocytogenes*	CH	LHICA 4	32	32	16	32	32
*L. monocytogenes*	CH	LHICA 5	16	16	32	32	32
*L. monocytogenes*	FP	LHICA 6	16	16	16	32	32
*L. monocytogenes*	FP	LHICA 7	32	32	32	16	32
*L. monocytogenes*	CC	CECT 934	32	16	32	16	32
			n (%)	n (%)	n (%)	n (%)	n (%)
MIC (µg/mL)		16	4	6	2	4	0
		32	4	2	6	4	8

CC, culture collection; CH, cheese; FP, fish product; RM, rabbit meat.
